# A Comparative Study of Different Administrations of Nebulized Hyaluronic Acid After Endoscopic Endonasal Surgery for Chronic Rhinosinusitis

**DOI:** 10.1007/s12070-020-02110-6

**Published:** 2020-09-02

**Authors:** Vincenzo Abbate, Giorgio Iaconetta, Fabio Maglitto, Giovanni Improta, Antonio Romano, Paola Bonavolontà, Francesco Seidita, Luigi Califano, Giovanni Dell’Aversana Orabona

**Affiliations:** 1 Maxillofacial Surgery Unit, Department of Neurosciences, Reproductive and Odontostomatological Sciences, University Federico II, Via Pansini 5, 80100 Naples, Italy; 2 Neurosurgery Unit, Department of Medicine, Surgery and Odontoiatrics, University of Salerno, Via Giovanni Paolo II 132, 84084 Fisciano, Salerno Italy; 3 Department of Public Health, University of Naples ‘‘Federico II’’, Naples, Italy

**Keywords:** Hyaluronic acid, Nasal douche, FESS, ESS, SNOT-22, Nasal spray

## Abstract

Hyaluronic Acid (HA) plays many roles in wound healing in general, through different mechanisms. Several authors reported the effectiveness of hyaluronic acid in promoting mucosal healing and reducing discomfort for patients after endoscopic sinus surgery (ESS). Different methods for HA nasal administration have been reported. The aim of our study has been to evaluate the efficacy of the administration of nebulized HA through a nasal douche compared with its administration through a nasal spray with patients undergoing ESS for chronic rhinosinusitis. From January 2013 to January 2019 a prospective clinical trial was carried out in our hospital with 163 patients who had undergone ESS for chronic rhinosinusitis. The sample was divided into three groups according to the method of administration of HA. Our study confirm the efficacy of the administration of nebulized HA through nasal douche in post-operative care (6.5% vs 4.5%). The most relevant data regards the nasal dryness sign: the data revealed an unexpected percentage of worsening of that sign at time T3 (*p* = 0.049) particularly evident in the patients treated with HA through nasal douche compared to whom the nasal spray device was prescribed (4% vs 1%). Further studies are needed to identify the best means of administration of HA, which would satisfy the requirements for efficacy in terms of the results and, at the same time, patient compliance.

## Introduction

Hyaluronic acid (HA) is a carbohydrate, more specifically a high molecular weight mucopolysaccharide with a linear and poly repeating disaccharide structure. First discovered in 1934 (K. Meyer and J. Palmer), HA plays an important role in the formation of the extracellular matrix and in intracellular environment. The viscoelastic nature of HA, added to its biocompatibility and non-immunogenicity, has led to its use in various clinical applications in different medical fields such as dermatology, aesthetic medicine, ophthalmology, joint surgery and tissue engineering [1].

The main biological functions of HA are: the maintenance of the connective fluid tissue consistency and elastoviscosity features; tissue hydration control; water molecule carriage; and extracellular matrix proteoglycan assembly. It also has a role as a receptor in different processes such as inflammation, tumors development, mitosis and cell migration [2].

The role of HA in tissue regeneration and wound healing is well known. It has the capability to induce fibroblast stimulation and collagen deposition.

Several authors have described the use of HA with a nasal administration following endoscopic endonasal surgery (EES) and its effectiveness in improving the endonasal mucosal membrane healing [3–6].

Different methods for HA nasal administration have been reported: nebulized through sprays or ampoules, or topically administered with cream or nasal packing [3, 6].

These authors have confirmed the efficacy of the administration of nebulized HA through a nasal douche, compared with saline solution alone; but still now there are no comparative studies evaluating the efficacy of nebulized HA administered through nasal sprays in patients undergoing EES.

Actually, even if the intake of HA through nasal douche, is more effective, it is not equally accepted by the patient due to the higher costs and longer times required for administration compared to a pocket spray device

The aim of our study has been to evaluate the efficacy of the administration of nebulized HA through a nasal douche compared with its administration through a nasal spray with patients undergoing EES for chronic rhinosinusitis (CRS).

## Methods

From January 2013 to January 2019 a prospective clinical study was carried out in the Maxillofacial Surgery Unit of the University of Naples Federico II with patients who had undergone ESS for CRS.

One hundred Sixty-three (163) patients met the following criteria and were enrolled in the study. The inclusion criteria were: CRS; and a 12-month follow-up. The exclusion criteria were: previus oncological disease; no previous surgery; and acute rhinosinusitis.

All the enrolled patients underwent endoscopic surgery (functional ESS and/or ESS) at our Department. All of them were operated on by the same surgical team and received pharmacological therapy, based on corticosteroids and antibiotics for 10 days after surgery.

After nasal packing removal, our sample was divided into three groups based on the modality of post-surgical treatment. Each patient was randomly assigned by a computerized system to one of the three groups.

The patients in group A were treated with the administration of nebulized HA through a nasal douche (*Rinowash* nasal douche, © Air Liquide Medical Systems, Paris, France) twice a day for 30 days; the patients in group B were treated with the administration of nebulized HA through a common nasal spray device twice a day for 30 days; and, finally, the patients in group C (the control group) were treated with a saline solution alone administered by a syringe daily for 30 days. (Table 1)

**Table 1:**
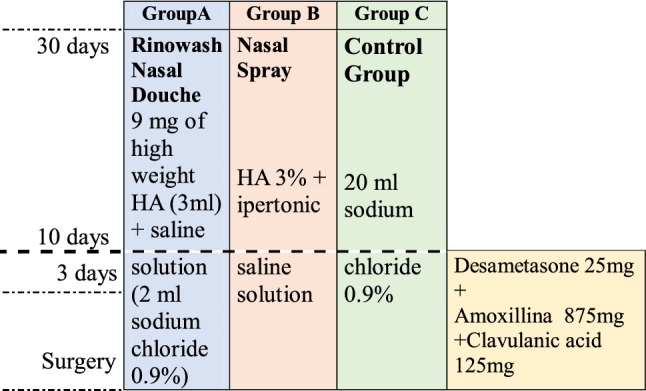
Postoperative therapeutic protocol applied to the three groups

Before undergoing surgery (T0), and at the two week follow-up (T1), four week follow-up (T2), and one year follow-up (T3) the patients from all three groups were clinically and endoscopically examined by means of two techniques: first, via nasal fibro endoscopy, which allowed different signs (nasal dryness, mucosal hyperemia, mucosal edema, secretions, crusting, traces of blood, synechia and polyps) to be studied according to a dichotomous scale (i.e. presence/absence); and secondly, a self-assessment questionnaire, the *Sino*-*Nasal Outcome Test (SNOT*-*22),* to report a subjective assessment of the post-operative quality of life and post-operative speed of recovery.

All the patients studied signed an informed consent approved by the Local Ethics Committee in compliance with the regulations in force.

### Statistical analysis

The reported data were collected and analyzed by using Microsoft Excel 2018 to calculate medians, means and the relative standard deviations (SD) where appropriate. In addition, the Statistical Package for Social Sciences (SPSS) was used for the Shapiro–Wilk normality test and for the test of the medians with independent samples in which a *p* value < 0.05 was considered statistically significant. The average and median of the tabulated values were calculated in relation to the endoscopic signs, with the SD calculated by group considering the mean and median for the different parameters at the different time points (T_0_, T_1_, T_2_ and T_3_).

The null-hypothesis of the Shapiro–Wilk test was normally distributed.

## Results

The samples were randomly divided into the three groups. Among the 163 patients, 99 were male and 64 were female, aged between 21 and 72 years, with an average age of 53. Sixty-five patients (39.9%) had been affected by maxillary sinusitis (monolateral or bilateral), 45 (27.6%) by ethmoid-maxillary sinusitis, 38 (23.3%) by fronto-ethmoid-maxillary sinusitis and 15 (9.2%) by pansinusitis. 97 (59.5%) had also been affected by nasal septum deviation and/or hypertrophy of the turbinates (Table 2).

**Table 2:** Percentage of pathology divided by location on our samples

Disease	Number of patient (total 163)	%
Maxillary sinusitis	65	39.8773
Ethmoido-maxillary sinusitis	45	27.60736
Fronto-etmoido-maxillary sinusitis	38	23.31288
Pansinusitis	15	9.202454
Nasal septum deviation and/or hypertrophy of the turbinates	97	59.5092

The analysis of the scores resulting from the SNOT-22 showed a significant improvement in the symptomatology of group A at the time T1.

**Fig. 1:**
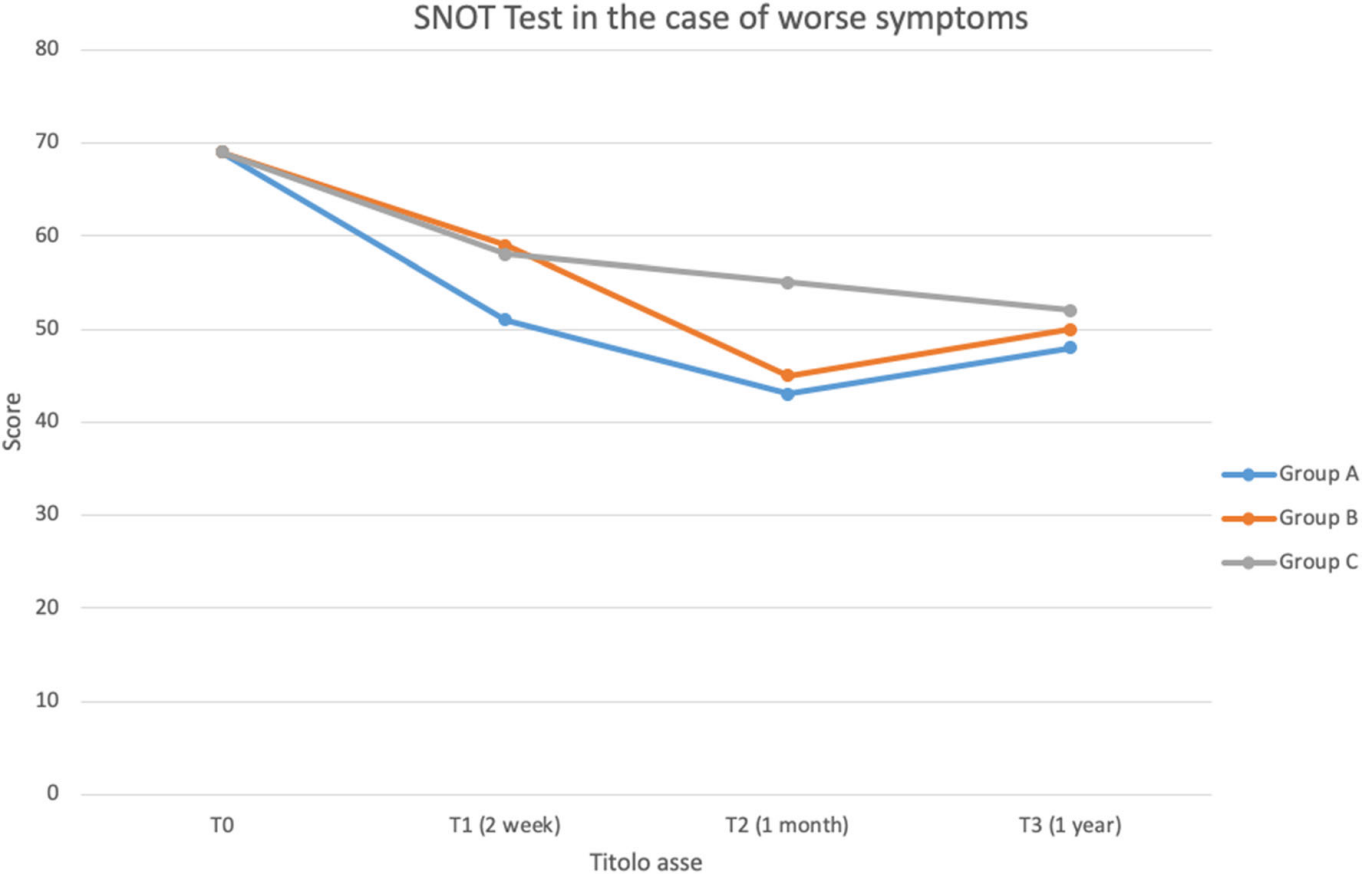
SNOT test in the case of worse symptoms

At the time T2, group B achieved a progressive improvement comparable to that of group A. At the one-year follow-up (T3), the scores of the three groups overlapped within a narrow range of values (Fig. 1).

As regards the clinical signs, evaluated by means of the nasal endoscopy, the analysis of the average of the values found, showed an overall improvement in all three groups from times T1 to T3, with a trend superimposable to that observed by the SNOT-22.

**Fig. 2:**
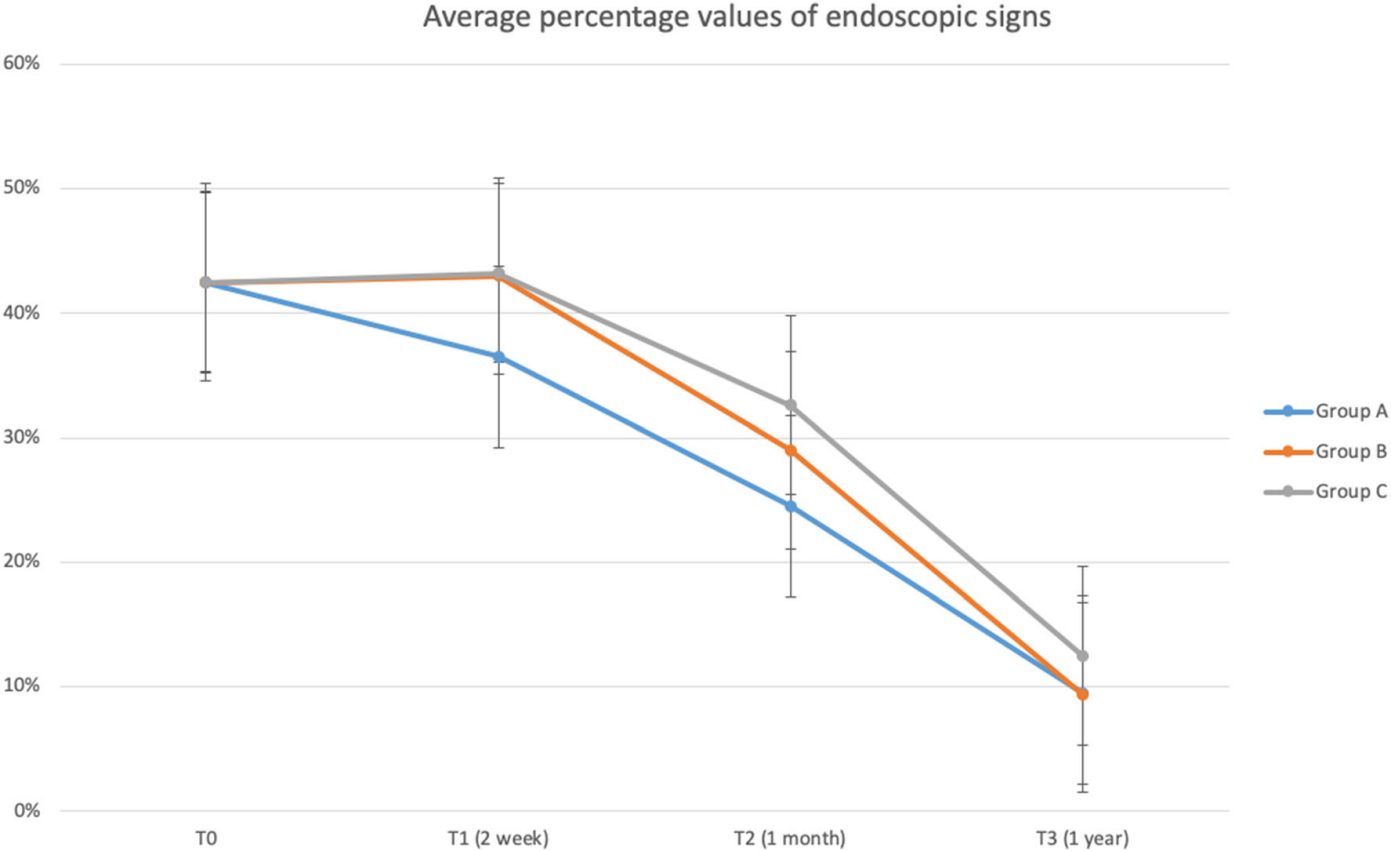
Average percentage values of endoscopic signs detected at times T0, T1, T2, T3

In particular, group A showed an improvement in symptoms already at times T1 and T2, the incidence reducing from 43 to 36.5% and 24.5%; group B showed an overall improvement at T3 comparable to that of group A (9.38% vs 9.50%) but with a later response. Group C showed a slightly lower symptom resolution at time T3 than the other groups (12.5% vs 9.38% vs 9.5%) with a slower recovery trend (Fig. 2).

Analyzing the individual endoscopic signs, it was possible to highlight how for each symptom there was an overall improvement at time T3 with a more favorable trend in terms of resolution of the symptomatology for groups A and B.

**Fig. 3:**
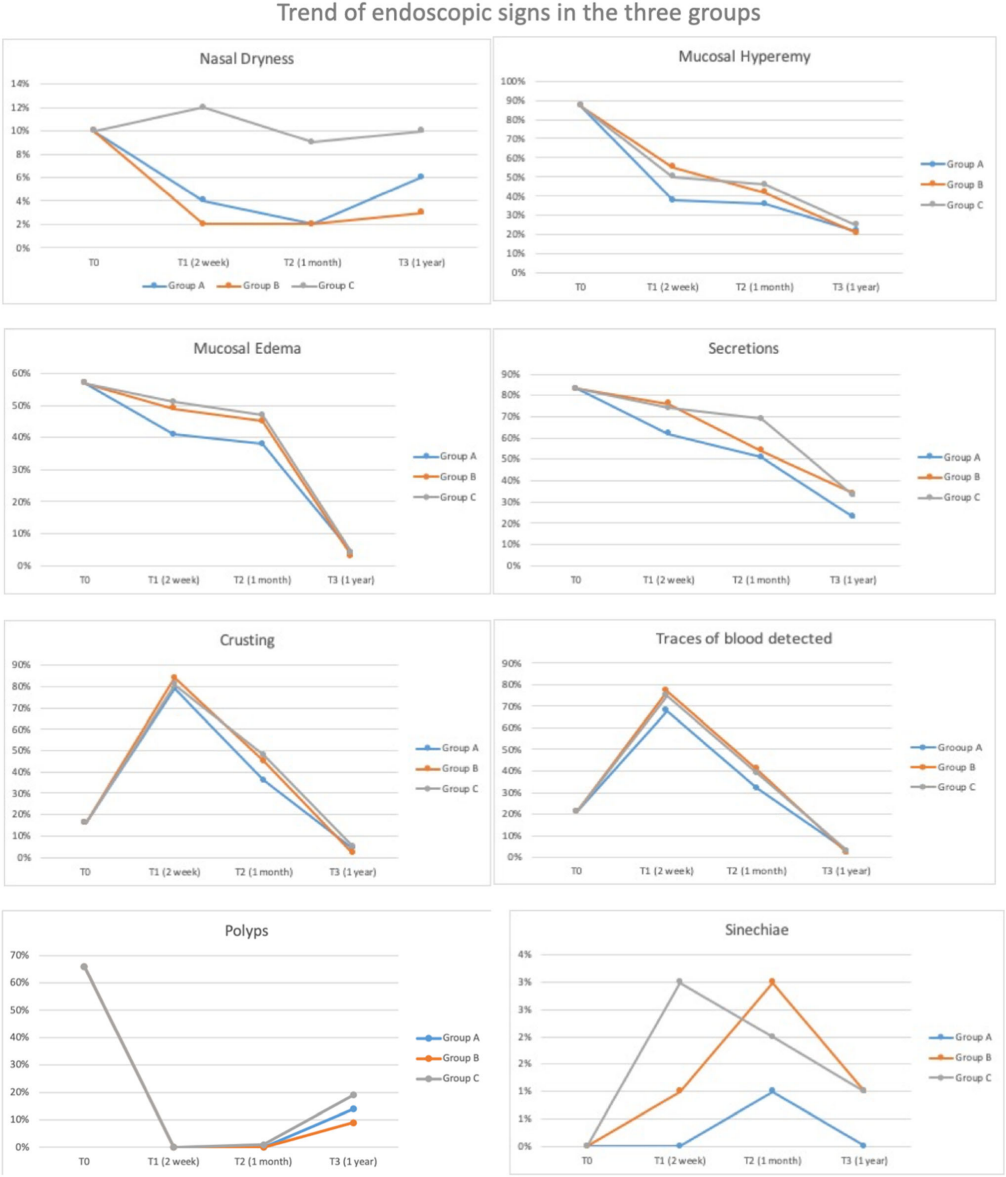
Trend of endoscopic signs in the three groups

With regard to nasal dryness, a considerable discrepancy of the percentage values at time T3 in the three groups was highlighted. In particular, the use of HA in the form of a nasal douche or nasal spray (Groups A and B, respectively) significantly reduced nasal dryness at times T1 and T2 (*p* = 0.049), however showing a reversal trend at time T3 greater in group A (6% vs 3%) (Fig. 3; Table 3).

**Table 3:** Normality test for each endoscopic sign

		Shapiro–Wilk		
		Statistica	*df*	Sig. (*p* value)
Nasal dryness	Group A	0.97	4.00	0.049*
	Group B	0.72	4.00	
	Group C	0.89	4.00	
Mucosal hyperemy	Group A	0.84	4.00	0.37
	Group B	0.98	4.00	
	Group C	0.94	4.00	
Mucosal edema	Group A	0.92	4.00	0.368
	Group B	0.81	4.00	
	Group C	0.78	4.00	
Secretions	Group A	0.99	4.00	0.368
	Group B	0.94	4.00	
	Group C	0.86	4.00	
Crusting	Group A	0.93	4.00	1.00
	Group B	0.95	4.00	
	Group C	0.94	4.00	
Traces of blood	Group A	0.96	4.00	1.00
	Group B	0.98	4.00	
	Group C	0.97	4.00	
Sinechiae	Group A	0.63	4.00	0.264
	Group B	0.89	4.00	
	Group C	0.99	4.00	
Polyps	Group A	0.77	4.00	1.00
	Group B	0.72	4.00	
	Group C	0.81	4.00	

*Statistically significant *p* value < 0.05

## Discussion

Post-operative care in endoscopic nasal and endosinusal surgery is recognized to be mandatory for an optimal healing, to limit secretions and crusting and to prevent the formation of polyps [7–9].

Frequent nasal washings are a very important component of post-operative care. These are generally performed with isotonic or hypertonic solutions, with a different osmolality. The most common is saline solution with 0.9% sodium chloride [10]. Recently, due to evidence of the beneficial impact of HA on mucosal healing, the tendency to use HA-based products for nasal washes has been increasing [11].

HA is a glycosaminoglycan, a linear and anionic polymer consisting of two linked sugars, glucuronic acid and N-acetylglucosamine. It tends to have a gelatinous physical state and has the capability to hydrate tissues due to its ability to bind water molecules. It is one of the main components of the extracellular matrix. Its chemical-physical features are responsible for its high biocompatibility and absorbability.

HA plays many roles in wound healing in general, through different mechanisms. The extracellular matrix rich in HA tends to stimulate cell migration by increasing both cell motility and proliferation. It also acts as a signal molecule for cell migration and induces neo-angiogenesis due to the inflammatory environments resulting from its degradation [12–17].

HA plays an important role in nasal mucosal healing. When applied, it creates a gelatinous layer over the intranasal surfaces and reduces the risk of synechia, providing an adequate mucosal hydration and vascularization. Furthermore, Cassano et al. [18] reported that in patients undergoing EES, HA promoted both mucociliary clearance and mucosal regeneration as a consequence of a faster recovery of the impaired ciliated cells. The action of HA on the ciliary motility may be explained by the fact that the breaking of each molecule, initiated by free radicals and hyaluronidase during inflammation, into active fragments with a low molecular weight increases the frequency of the ciliary beat. This action is mediated by the increase in the concentration of calcium ions and the interactions between the HA fragments, HA-mediated motility receptors and receptors of origin are present on the cilia [19]. Thus, HA limits the incidence of rhinorrhea and nasal obstruction, and reduces the number of secretions on endoscopic evaluation due to improvements in the mucociliary clearance [20].

Several Authors [4, 5] have reported the beneficial healing effect of the administration of nebulized HA on the nasal mucosa, in comparison with the use of saline solution alone, after EES [21]. Based on our clinical experience, nasal douche requires a high level of patient compliance due to the time required for the preparation of the therapy and the need for an electric nebulization device. For this reason, we have evaluated the efficacy of the administration of nebulized HA through a nasal spray, comparing it with that achieved by using a nasal douche. These nasal sprays are pocket-sized, ready-to-use and, therefore, are generally tolerated better by the patient.

Our results have confirmed the efficacy of the HA administration through a nasal douche, especially in terms of the speed of healing at time T1. As reported in our results, we have revealed a rapid improvement in the symptoms detected during the clinical examination in the group A, compared to the group B where the administration was performed through a nasal spray, with a percentage of improvement of about 6.5% at T1 and 4.5% at T2.

Nevertheless, at times T2 and T3 the difference between the two groups (A and B) is greatly reduced (Figs. 1, 2). The most relevant data regards the nasal dryness sign: The data show a statistically significant improvement of the sign (*p* = 0.049) in the groups A and B at times T1 and T2 with an unexpected percentage of worsening of the sign at time T3 particularly evident in group A compared to B (4% vs 1%).

This evidence may be linked to a lower long-term tolerability in the administration of HA through the nasal douche compared to a more manageable long-term administration using a nasal spray.

In our case series, even though it is better tolerated, the administration through a spray has proved less effective in improving the post-operative symptomatology. Casale et al. [22–24] described a new nasal device for the administration of nebulized HA. The results of this prospective study suggest the administration of nebulized HA through this new device (Spray-sol) as a supportive treatment for a faster improvement of nasal respiration, also minimizing patient discomfort and promoting nasal mucosa healing in post-operative ESS for CRS.

The results of our study confirm the efficacy of nebulized HA in post-operative treatment. In order to reduce any complications and to obtain a faster nasal mucosa healing nebulized HA is recommended. We can observe from our study a better patient compliance when a pocket device is used.

Further studies are needed to identify the best means of HA administration, which would satisfy the requirements for an efficacy in terms of the results and, at the same time, patient compliance.
